# The assessment of cadmium, chromium, copper, and nickel tolerance and bioaccumulation by shrub plant *Tetraena qataranse*

**DOI:** 10.1038/s41598-019-42029-9

**Published:** 2019-04-04

**Authors:** Kamal Usman, Mohammad A. Al-Ghouti, Mohammed H. Abu-Dieyeh

**Affiliations:** Department of Biological & Environmental Sciences, College of Arts & Sciences, Qatar University, Doha, Qatar

## Abstract

Heavy metals constitute some of the most significant environmental contaminants today. The abundance of naturally growing *Tetraena qataranse* around Ras Laffan oil and gas facilities in the state of Qatar reflects its toxitolerant character. This study examined the desert plant’s tolerance to Ba, Cd, Cr, Cu, Ni and Pb relative to soil concentration. Analysis by inductively coupled plasma – optical emission spectroscopy (ICP-OES) showed that the plant biomass accumulates higher Cd, Cr, Cu and Ni concentration than the soil, particularly in the root. The bioconcentration factor (BCF) of all metals in the root and shoot indicates the plant’s capacity to accumulate these metals. Cd had a translocation factor (TF) greater than one; however, it is less than one for all other metals, suggesting that the plant remediate Cd by phytoextraction, where it accumulates in the shoot and Cr, Cu and Ni through phytostabilization, concentrating the metals in the root. Metals phytostabilization restrict transport, shield animals from toxic species ingestion, and consequently prevent transmission across the food chain. Fourier Transform Infrared Spectroscopy (FTIR) analysis further corroborates ICP-OES quantitative data. Our results suggest that *T. qataranse* is tolerant of Cd, Cr, Cu, and Ni. Potentially, these metals can accumulate at higher concentration than shown here; hence, *T. qataranse* is a suitable candidate for toxic metals phytostabilization.

## Introduction

Heavy metals are some of the most significant environmental contaminants; this is in large part due to anthropogenic activities arising from industrialization^1^. Though physical and chemical treatment strategies to remove metal pollutants exist, such methods are labor intensive. Additionally, chemical treatment methods are expensive and generates other pollutants^2^. Therefore, the need for alternative technologies became imperative and the exploration of different bio-based techniques, i.e. bioremediation, ensued. The use of biological agents are cheap, safer, and has limited or no adverse effects on the environment. Bioremediation methods include bio-augmentation, bioremediation, bioventing, composting and phytoremediation. Of these, phytoremediation proves the most viable and cheaper alternative and has since gained increased attention^3^.

The use of plants, such as *T. caerulescens* and *V. calaminaria*, to remove heavy metal dates back hundreds of years^4^. Many studies suggest that the accumulation of heavy metals in plant tissues could lead to the inhibition of the plant’s significant enzymatic activity resulting in a wide range of adverse effects on germinability, seedlings development, and photosynthetic processes^5^. Under heavy metal stress, plants appear to exhibit increased production of reactive oxygen species (ROS). As a result; some plants have developed mechanisms to counteract the effects of this stress condition. One of these mechanisms is the increased activity of specific antioxidant enzymes, such as peroxidase (POD), catalase (CAT) and superoxide dismutase (SOD)^6^. The root, essential to plant development, plays a vital role in how they respond to stress including stress due to heavy metal exposure. The root cell wall has a mechanism of exchange that fixes heavy metal ions and limits the transmission of toxic effects to other tissues^7,8^.

Several parameters, including bioconcentration factor (BCF) and translocation factor (TF) can be used to evaluate plant phytoremediation potential. A BCF value higher than one indicates that a plant is a hyperaccumulator, whereas a value less than one is indicative of an excluder. TF value determines plant efficiency in heavy metals translocation from the root to the shoot. A plant is considered efficient in metal translocation from root to shoot when TF is higher than one; this is due to an efficient metal transport system. TF values less than one, however, indicate ineffective metal transfer suggesting that these type of plants accumulate metals in the roots and rhizomes more than in shoots or the leaves^9^.

Present in the plant are macromolecules, including carbohydrates, lipids, and nucleic acids that bear distinct functional groups that interact with transition metals. These functional groups correspond to specific infrared light frequencies^10^ and their interactions with these metals can be analyzed by Fourier Transformed Infrared Spectroscopy (FTIR). Therefore, FTIR data can be used to quantitatively determine the heavy metal presence in plant tissues via metal cation binding in plant samples^11^.

**Figure 1:**
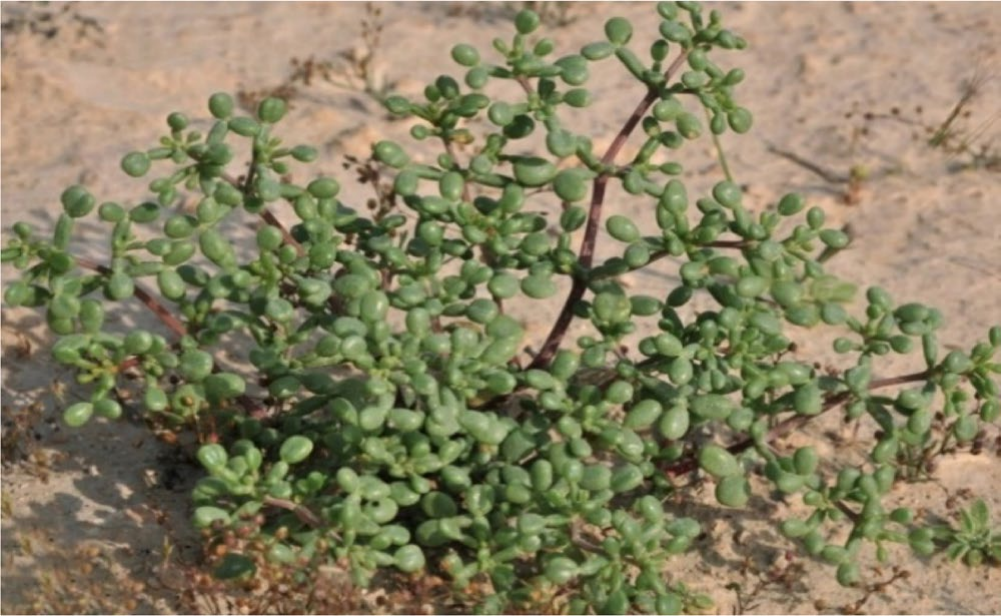
Naturally growing *Tetraena qataranse* at Ras Laffan Industrial area, Qatar.

The metals, Ba, Cd, Cr, Cu, Ni and Pb, pose significant public health risks and evidence of frequent occurrence in the Qatari environment is well documented in the literature^1,12^. Phytostabilization, a form of phytoremediation, allows plants to stabilize metals and to reduce their presence in water percolating through the soil matrix. This limits the formation of toxic and dangerous leachates^13^. Several plants species, such as *F. rubra* L. are reported to be useful in the process of phytostabilization of heavy metals in the soil^14^. *T. qataranse* is one of the most common desert plants naturally growing in Ras Laffan area. A desert undershrub, well adapted to rocky and sandy saline soil, it is characterized by fleshy terminal branches and succulent leaves (Fig. 1)^15^.

In this study, we assess heavy metals (Ba, Cd, Cr, Cu, Ni and Pb) accumulation in four plant species (*T. qataranse, S. vermiculata*, *L. axillare*, and *S. aegyptiaca*)) growing in an industrial area with high anthropogenic activities (Ras Laffan) in the state of Qatar. Following metal quantitation, the most promising species (*T. qataranse)* potentials for toxic metals phytostabilization is evaluated. Inductively Coupled Plasma-Optical Emission Spectroscopy (ICP-OES) and FTIR data detect the presence of heavy metals in *T. qataranse* tissues after exposure. Here, we show that high metal accumulation by *T. qataranse* makes it a suitable candidate for toxic metals phytostabilization.

## Results

### Soil properties

Except for the toxic metals, the soil properties at Ras Laffan is generally similar to that of Qatar University, hence its choice as the control area to compare FTIR data. Analysis of Ras Laffan soil gives a pH of 8.05 ± 0.12, an electrical conductivity of 4.25 ± 0.44 mS/m and 1.1 ± 0.03% TOC level, revealing its alkaline and saline nature. As for the control site, pH level stood at pH of 8.31 ± 0.36, an electrical conductivity of 4.63 ± 0.61 mS/m and 0.57 ± 0.01% TOC. No differences in the pH and EC in the soil from the control site (Qatar University). Qatari soil is indeed mostly alkaline with high calcium-magnesium carbonates composition. It has relatively low organic matter contents as well as significant iron and clay constituents^12^.

### Heavy metal bioaccumulation

**Figure 2:**
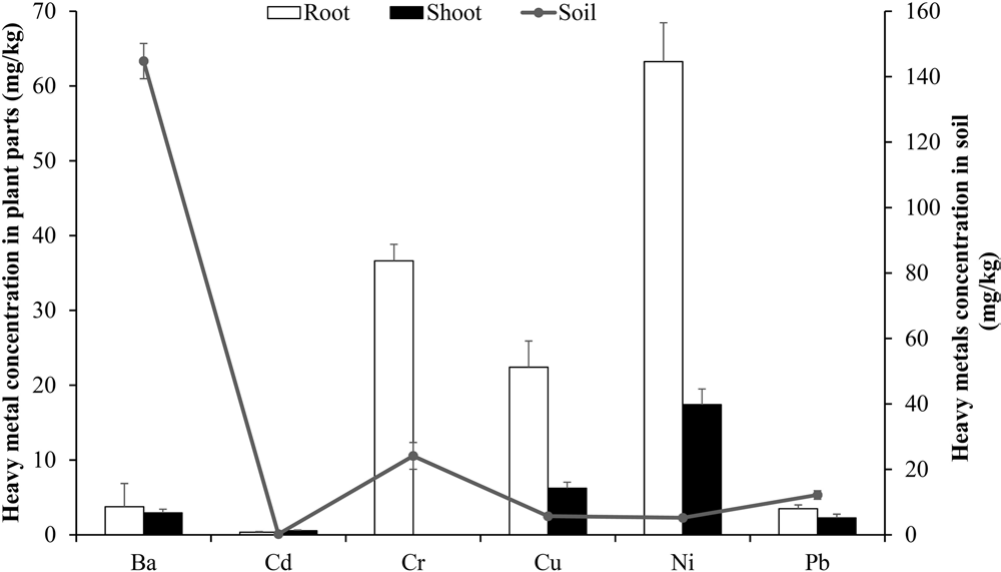
Heavy metals concentration in the soil, root and shoot of *T. qataranse*. Mean concentration of metals are averages of five replicates (n = 5) ± SEM at *P* < 0.05 level.

Several desert plants are tolerant to heavy metals stress. Some of these are commonly found growing on Qatari soil; examples include *P. australis, T. domingensis*, *P. juliflora*, *Tamarix spp*., *M. polymorpha* and others^1,16^. In a preliminary study, we found that *T. qataranse* grows when irrigated with oil and gas produced water containing different heavy metals at varying concentrations^17^. However, to the best of our knowledge, this is the first study examining *T. qataranse* specific metal tolerance and tissue accumulation capacity relative to background metal concentration in the soil. Metals concentration were also determined for other plant species (*S. aegyptiaca, S. vermiculata* and *L. axillare*) found growing in the same area. However, tissue accumulations by these plants were much lower (see Supplementary Fig. S1a–c), and therefore, this work discuss *T. qataranse* results, only. Metal concentration in the soil and plant tissue parts (Root and shoot) is as shown in Fig. 2. Soil concentration (mg/kg) dry weight are in the following order Ba (144.8) > Cr (24.1) > Pb(12.2) > Cu (5.7) > Ni (5.19) > Cd (0.2) mg/kg. Although industrial activities increase toxic metals contamination, soil leaching or wearing reduces metal concentration in affected areas^18^. For the plant tissue parts (root and shoot), metal accumulation follows the trend Ni (63.3) > Cr (36.6) > Cu (22.4) > Ba (3.8) > Pb (3.5) > Cd (0.4) and Ni (17.4) > Cu (6.2) > Ba (2.9) > Pb (2.3) > Cd (0.5) > Cr (0) mg/kg respectively. It is important to note that Ba, Cr and Pb concentrations in the soil are much higher than that of Cd, Cu and Ni (Fig. 2). However, accumulation of these metals in *T. qataranse* tissues was not impressive, except for Ni and Cr with up to 63.3 and 36.6 mg/kg in the root, respectively. Contrastingly, Cr was found to be below the detection limit in the shooting part, whereas Ni accumulates up to 17.4 mg/kg in the shoot. Soil properties, which in turn determines metal bioavailability for plant uptake is partly responsible for the non-translocation of Cr. Other vital factors include metal behavior and toxicity to the plant species in question^19^.

### Correlation analysis

The phytoavailability of heavy metals in the soil can be determined by establishing a correlation between metal concentration in the soil and accumulation in plant tissues^20^, subject to plant type and soil properties, especially elemental composition^21^. Single regression analysis is used to assess the phytoavailability of all analyzed metals in this study. The correlation coefficients (*r*) of various metals were computed using Pearson’s regression equation at two levels (i) between metal concentration in the soil and different plant parts (root and shoot), and (ii) between metal concentration in the root and shoot. The correlation coefficient was used to determine positive or negative correlations, indicating the suitability or unsuitability of metal accumulation in plants. Table 1 shows a relationship between metals in the soil and root/shoot metal concentration in *T. qataranse* grown naturally in the soil. Correlation coefficient between metals in *T. qataranse* tissue parts (root and shoot) are as shown in Table 2.

**Table 1:** Correlation between metals in soil and plant parts of *T. qataranse*.

Metals (*n* = 5)	Plant parts	
	Root	Shoot
Ba	−0.66	−0.98^*^
Cd	0.02	0.28
Cr	0.60	n.a
Cu	−0.11	−0.75
Ni	0.96^*^	0.45
Pb	0.76	−1.00^*^

*Correlations is significant at 0.05 level.

n.a = Correlation data not applicable (No metal accumulation in the shoot).

**Table 2:** The correlation coefficient (r) between root and shoot metal concentration in *T. qataranse* (*n* = 5).

Shoot	Ba	Cd	Cr	Cu	Ni	Pb
Ba	0.50	−0.88	−0.71	−1.00^*^	−0.86	−0.50
Cd	−0.28	−0.95	−1.00^*^	−0.68	−0.96	0.28
Cr	n.a	n.a	n.a	n.a	n.a	n.a
Cu	−0.39	−0.91	−0.99^*^	−0.58	−0.93	0.39
Ni	−0.98^*^	0.21	−0.07	0.67	0.18	0.98^*^
Pb	0.79	0.60	0.80	0.12	0.63	−0.79

*Correlations are at 0.05 significance level.

n.a = Correlation data not applicable (No metal accumulation in the shoot).

### Bioconcentration factor (BCF) and translocation factors (TF)

**Figure 3:**
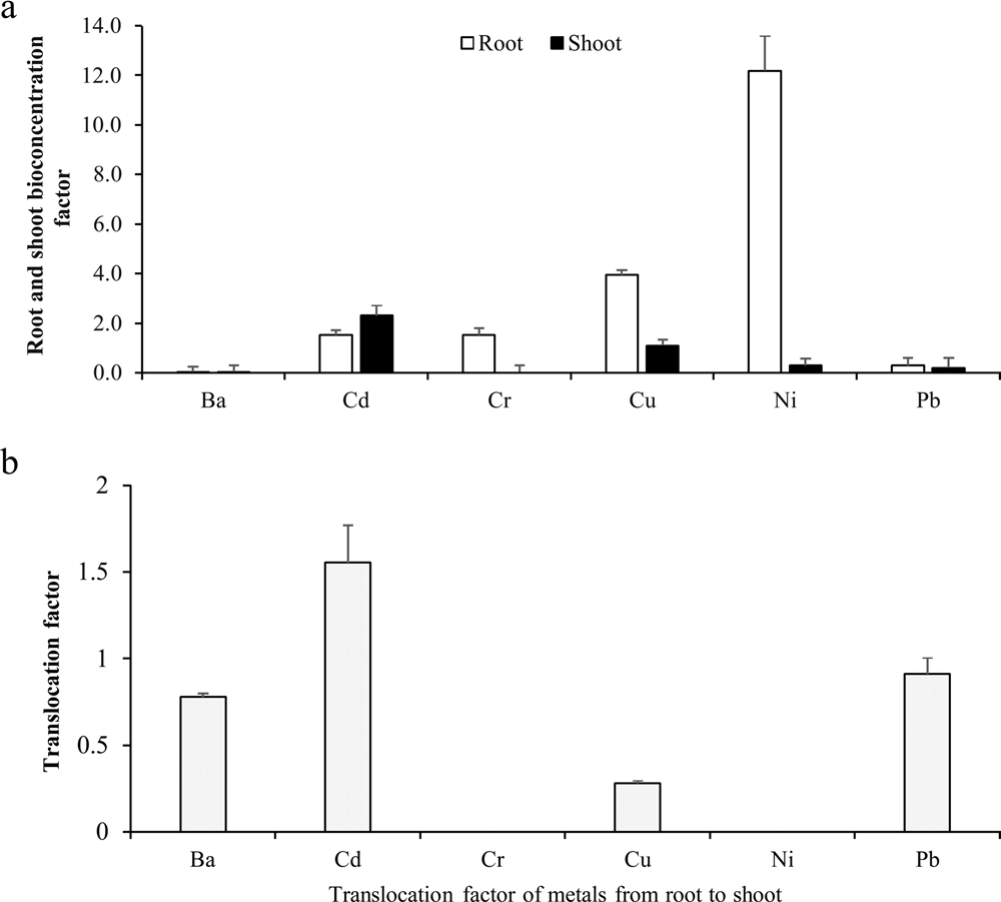
Bioconcentration factor (**a**) and translocation factor (**b**) in *T. qataranse* tissues. Mean plant tissues BCF are averages of five BCF values (n = 5) ± SEM.

Bioconcentration (BCF) and translocation (TF) factors are important parameters used in the feasibility study of heavy metals plants remediation potential, phytoremediation^13^. The evaluation of *T. qataranse* accumulation of Ba, Cd, Cr, Cu, Ni and Pb by approximate BCF and TF are shown in Fig. 3a,b respectively. The BCF values for Cd, Cr, Cu and Ni are greater than one, but lower than one for Ba and Pb. Root BCF were in the order; Ni (12.2) > Cu (3.9) > Cd (1.5) > Cr (1.5) > Pb (0.2) > Ba (0.02), shoot BCF are Ni (3.3) > Cd (2.3) > Cu (1.1) > Pb (0.2) > Ba (0.02) > Cr (0.0) mg/kg. Together, the BCF values indicates Cd, Cr, Cu and Ni phytostabilization by *T. qataranse*. On the other hand, only Cd had a TF greater than one at 1.6 (Fig. 3b), while the TF of all other metals (Ba, Cr, Cu, Ni and Pb) is less than one, suggesting *T. qataranse* Cd phytoextraction.

### FTIR analysis

**Figure 4:**
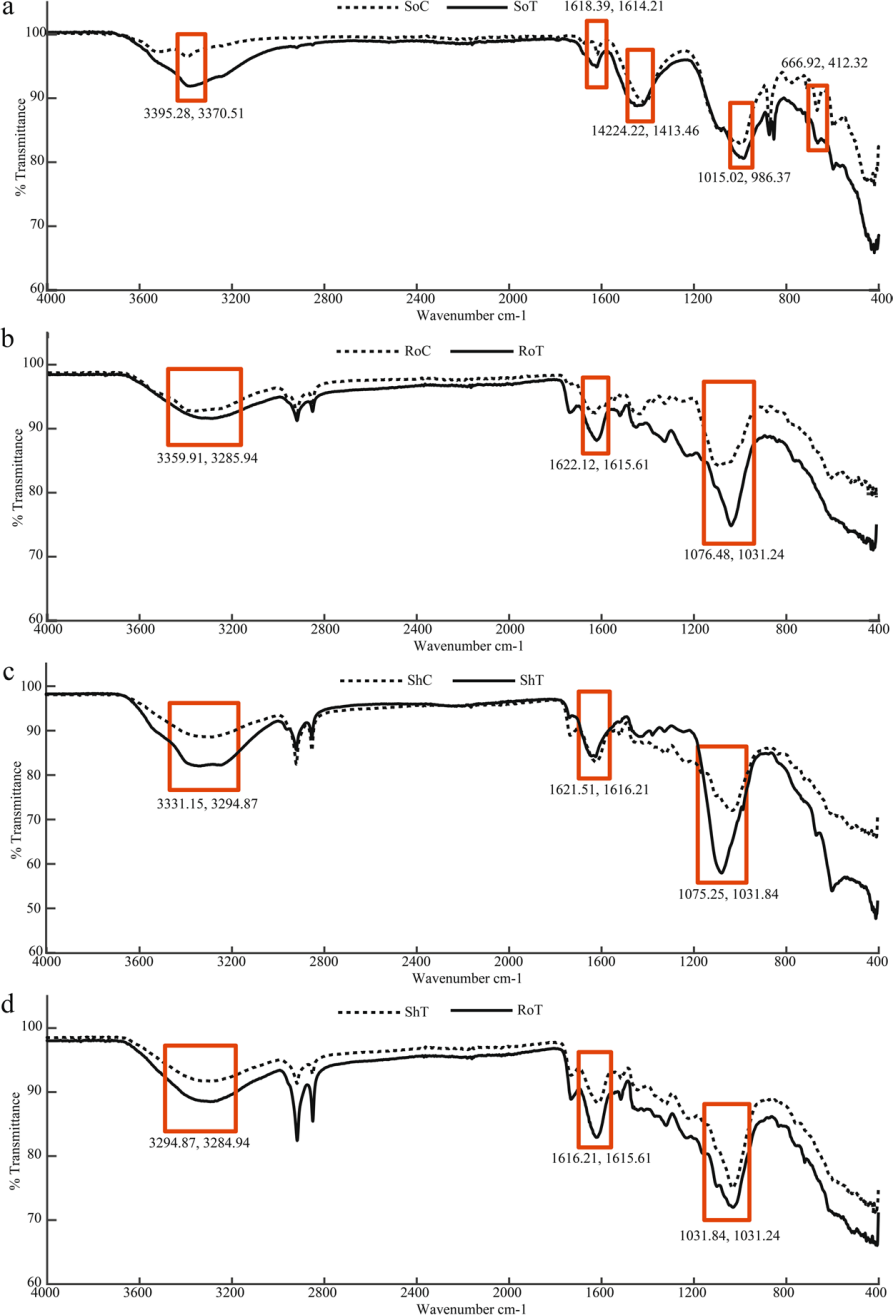
FTIR spectra for soil (**a**) and *T. qataranse* root and foliar (**b**–**d**). (**a**) Soil control (SoC) vs Soil treatment (SoT). (**b**) Root control (RoC) vs Root treatment (RoT). (**c**) Soot control (ShC) Shoot treatment (ShT). (**d**) Root treatment (RoT) vs Shoot treatment (ShT).

Transition metals found in biological samples interact with the functional groups of biomolecules, the composition of which can be determined by analysing their infrared light adsorption^11^. Therefore, FTIR data can be used to study metal cation binding in biological samples^22^. For FTIR analysis, soil and *T. qataranse* samples collected from a non-metal polluted area located at Qatar University as controls. All metals in the control sites were below detection limit when analyzed using ICP-OES. In general, there was no consistency in band shifts between treatment and control, especially in *T. qataranse* biomass. We recognize that since all samples analysed were obtained from the field, it is possible that other parameters have an influence on our FTIR result^23^. It is therefore important to note that, only where obvious (reported in the literature) is FTIR result used to infer metal presence on *T. qataranse* tissues, and to corroborate ICP-OES quantitative data. Although, up to five major bands were found to correspond to metal interaction in the soil (Fig. 4a), only three of these peaks, and their corresponding shifts were consistent on comparison to *T. qataranse* tissues, and therefore critically examined (Fig. 4b–d). FTIR results showed that dry biomass has different functional groups available for binding of heavy metal ions, such as carboxyl, phosphate, amide, and hydroxide. Broad and robust IR spectra regions spanning 3600–3200 cm^−1^ characterize O-H and N-H stretch^24^. All bands at 1618.39 and 1614.21 cm^−1^ (Fig. 4a), 1622.12 and 1615.61 cm^−1^ (Fig. 4b), 1621.51 and 1615.21 cm^−1^ (Fig. 4c) as well as 1616.184 and 1615.61 cm^−1^ (Fig. 4d) corresponds to specific amide groups due to C=O stretch^25^. The regions from 1200 to 900 cm^−1^ signify C-C, C-O, and C-O-P stretch overlaps^26^ occurring mainly in cellular polysaccharides.

## Discussion

Although few studies are available on soil heavy metal pollution in the state, Peng, *et al*.^12^ noted the spatial distribution of Cr, Cu, Ni and Pb on Qatari soil. The effects of toxic metals accumulation in the soil are not limited to inland environment and biota. Subject to their properties, metals travel across the soil environment, and subsequently deposits into water bodies. In Qatar, desalination is the main source of household water supply. A recent survey of household water quality in some Doha residences by Nriagu, *et al*.^27^ found the concentration of some toxic metals (Cd, Cr, Ni and Pb) to be significantly lower than the set limits for water quality in the state. Transport, accumulation and bioconcentration of toxic metals in the sea, presents a potential health risk to both humans, and aquatic life. Several factors including soil pH, organic, metal type, concentrations in the soil, plant type and age affects plant metal uptake^28–32^. Although Ba is the metal with the highest concentration (144.8 mg/kg) in the soil at Ras Laffan (Fig. 2). However, poor accumulation in the plant tissue parts was observed, particularly in the shoot, where only 2.9 mg/kg translocated. These could partly be due to the metal unavailability for *T. qataranse* uptake. Consistent with these findings, Kowalska, *et al*.^33^ found that less than 1% of Ba was bioavailable for plant uptake from the soil with 300 mg/kg Ba concentration. Although some plants, including *T. domingensis* and *C. papyrus* tolerate Ba at low to medium concentration. It is toxic to plants at high concentration. Few known plants including *I. cordifolia* and *V. densiflora*^34^ can tolerate relatively high concentrations. Information on Ba toxicity to humans remains to be adequately established. However, Kowalska, *et al*.^33^ noted high mortality rate of people aged 65 in communities with elevated Ba concentration, due to high cardiovascular and heart disease incidence^34,35^. Cr records higher concentration in the soil after Ba, at 24.1 mg/kg (Fig. 2). Although undetected in the shoot, it preferentially accumulates in the root, with a total concentration of 36.6 mg/kg (Fig. 2). Cr rarely occurs naturally, and only exist in trace amount even when produced by anthropogenic activities^36^. Despite accumulation in the root, limited translocation to the shoot is expected, irrespective of its available form. The soil at Ras Laffan is alkaline at 8.05, and therefore likely to have more of bioavailable Cr^6+^. Cr^6+^ is highly poisonous with strong oxidation potential, more mobile than Cr^3+^ and mostly associated with oxygen as chromate or dichromate ions. In plants, it easily translocates to the aerial parts than Cr^3+^, due to its water solubility, ability to penetrate complex physiological barriers and transformation capacity. The most common form of naturally occurring Cr is Cr^3+^, which complexes with organic matter in the soil environment and largely innocuous. It exists as either chromic oxides (Cr_2_O_3_), hydroxides (Cr (OH_3_)) or sulfates (Cr_2_ (SO_4_)_3_.12(H_2_O). Cr^3+^ has a higher affinity to cation exchange sites of the cell wall, and hence less likely to move across plant tissues in substantial quantity^37^.

With Pb, both root and shoot accumulates lower concentrations of 2.5 and 2.3 mg/kg, respectively, compared to the soil 12.2 mg/kg (Fig. 2). Although Pb does translocate to the aerial part of plants, several studies found that it preferentially accumulates in the root^38,39^. Translocation of metal to the aerial parts in plants is restricted in some species including Pb, courtesy of many factors noted above. Another limiting factor for Pb is the complex transport mechanisms involved. Regulation begins when the metal enters the root using apoplast via water streams and into the inner endodermis region. In the course of transport, negatively charged molecules in the cell wall such as pectin can immobilize Pb ions; others are plasma membrane accumulation or precipitation of Pb insoluble salts. Even more convincing is the fact that, since Pb may be trapped in the endodermis by the Casparian strip, it can resort to symplastic transport by which most of the isolated Pb is excreted out of the plant^38^. On the other hand, Cu and Ni exhibit similar accumulation pattern. They accumulate higher tissue concentration relative to that of the soil, respectively (Fig. 2). Additionally, both translocate even higher concentrations to the shoot than the soil, suggesting *T. qataranse* phytoextraction potential. Of all the metals reported in this study, Ni showed higher accumulation in *T. qataranse* tissues, with a concentration of 63.3 and 17.4 mg/kg in the root and shoot, respectively (Fig. 2). Although it occurs in several forms, under natural environmental conditions, Ni is most commonly present in the oxidized state as N^2+^. Unlike Cd and Pb for instance, it is highly mobile and therefore easily transported from the soil unto the root and across other tissue parts. An extensive review of Ni accumulation and transport in plants, Amari, *et al*.^40^ noted several studies in which many plants demonstrate the capacity to uptake Ni^2+^ to different tissue parts, particularly at an alkaline pH level of 8 and above. Another important factor in plant metal uptake is the soil organic matter. Interestingly, plants root system influences soil organic matter, which in turn determines how it affects the mobility of the surrounding metals. In a recent study, Nguyen, *et al*.^41^ found a positive relationship between Ni and Cu availability and organic content level in plants rhizosphere. Cu is essential to plants growth and development. However, it can be toxic at a concentration of more than 20 mg/kg^41^. The same study found *P. arundinacea*, *T. repens* and *P. virgatum* to accumulate a higher concentration of Cu at 55.8, 41.8 and 29.4 mg/kg, respectively. As regards to Cd, it is the least concentrated in the soil at 0.2 mg/kg (Fig. 2). However, *T. qataranse* root and shoot Cd accumulation are higher relative to that of the soil with 0.4 and 0.5 mg/kg, respectively. Although not covered here, Fe and Zn are two elements known to have adverse effects on Cd uptake^42^. Zn inhibits Cd uptake by its higher affinity to a common transporter molecule across the root plasma membrane^43^. In both *T. qataranse* root and shoot, Cd concentrations are higher than that of the soil (Fig. 2). An explanation to this could be that either Fe or Zn was not available inconsiderable amount to deter Cd uptake, or their inhibition effect suppressed by other metals present. Equally important is the soil pH, which is one of the most critical factors affecting metal bioavailability in the soil. Many studies reported that Cd becomes less bioavailable in the soil with an increase in pH level^44,45^. The soil at Ras Laffan is alkaline, with a pH level of 8.05 ± 0.12, and may be responsible for the low bioavailable Cd in the soil. Indeed, several studies reviewed by Kirkham^46^ suggest pH level above 7.0 significantly reduce Cd bioavailability.

Correlation analysis between metals concentration in the soil, and *T. qataranse* root and shoot indicates a significant positive correlation between Ni concentration in the soil and that of the root part (*r* = 0.96, *p* < 0.05) (Table 1), implying that the root easily accumulates Ni. There is a highly significant, but negative correlation between the soil and shoot against Ba (*r* = −0.98, *p* < 0.05) and Pb concentration (*r* = −1.00, *p* < 0.05), respectively. The same significant correlation between metals concentration in *T. qataranse* root and shoot also exist (Table 2). There is a significant positive relationship between Pb concentration in the root against Ni concentration in the shoot (*r* = 0.98, *p* < 0.05). Contrarily, similar but negative correlation coefficient (*r* = −0.98, *p* < 0.05) exist between Ba concentration in the root against Ni shoot concentration. It is a known fact that certain elements like Cu^2+^ are essential to metabolic process in plants, but toxic when present in excess; it is, however, less toxic compared to non-essential elements like Cd and Pb^47^. There is no correlation between Cu concentrations in the soil and *T. qataranse*. Highly significant but negative correlation exists between root Cu concentration against Ba in the shoot (*r* = −1.00, *p* < 0.05). The same observation is made between Cr concentrations in the root and Cu (*r* = −0.99, *p* < 0.05), and Cd (*r* = −1.00, *p* < 0.05) concentrations in the shoot. BCF values indicates that the root accumulates more metals (Cr, Cu and Ni) than the shoot (Cd) (Fig. 3a). The translocation factor of these metals (Fig. 3b) showed that only Cd was transferred into the shoot. Indeed, numerous plants are known for reduced metal uptake to aerial parts, which preferentially accumulate in the root^39^. The BCF and TF suggest that *T. qataranse* is tolerant to Cd, Cr, Cu and Ni.

Examining our FTIR results, soil control (SoC) showed a band at 3395.28 cm^−1^, which is higher than that of soil treatment (SoT) at 3370.51 cm^−1^ (Fig. 4a). Similar trends were also observed for root control (RoC) at 3359.91 cm^−1^ shifting to 3285.94 cm^−1^ for root treatment (RoT) (Fig. 4b), from 3331.15 cm^−1^ for shoot control (ShC) to 3294.87 cm^−1^ for shoot treatment (ShT) (Fig. 4c), and finally, RoC at 3294.87 cm^−1^ decreasing to 3285.94 for RoT cm^−1^ (Fig. 4d). These band shifts are due to Cd^2+^, Cr^2+^, Cu^2+^, or Ni^2+^ cationic interaction with the hydroxyl group for metal oxygen binding. D’Souza, *et al*.^48^ noted a similar trend when *P. tetrastromatica* was treated with and without Cd. Similar pattern was observed by Panda, *et al*.^24^ following Cd and Ni adsorption by *L. sativus* biomass, and conclude that this binding interaction is between amide group and Ni^2+^ via nitrogen atom. Consistent with Al-Ghouti, *et al*.^49^, the relatively higher band shift in RoT to 1615.61 from 1622.12 cm^−1^ for RoC (Fig. 4b) is due to Cu^2+^ binding to a lignocellulose material in *T. qataranse* root biomass. There were sharp decrease in bands intensity for SoC at 1015.02 lowering to 986.47 cm^−1^ for SoT (Fig. 4a), RoC at 1076.48 to 1030.23 cm^−1^ for RoT (Fig. 4b) and ShC at 1075.25 to 1030.23 cm^−1^ for ShT (Fig. 4c). However, a narrower drop in intensity from 1031.84 to 1031.24 cm^−1^ for RoT against ShT (Fig. 4d) is observed. Both indicates shifts can be attributed to strong metal binding involving Cu^2+^, Cd^2+^ or Ni^2+^ ^50^. Finally, with consistent decrease in band intensity of RoT when compared to ShT (Fig. 4d), the FTIR further confirmed our ICP-OES result (Fig. 2), that *T. qataranse* adsorb more metals (Cr, Cu, and Ni) in the root, with Cd further translocating to the shoot.

**Figure 5:**
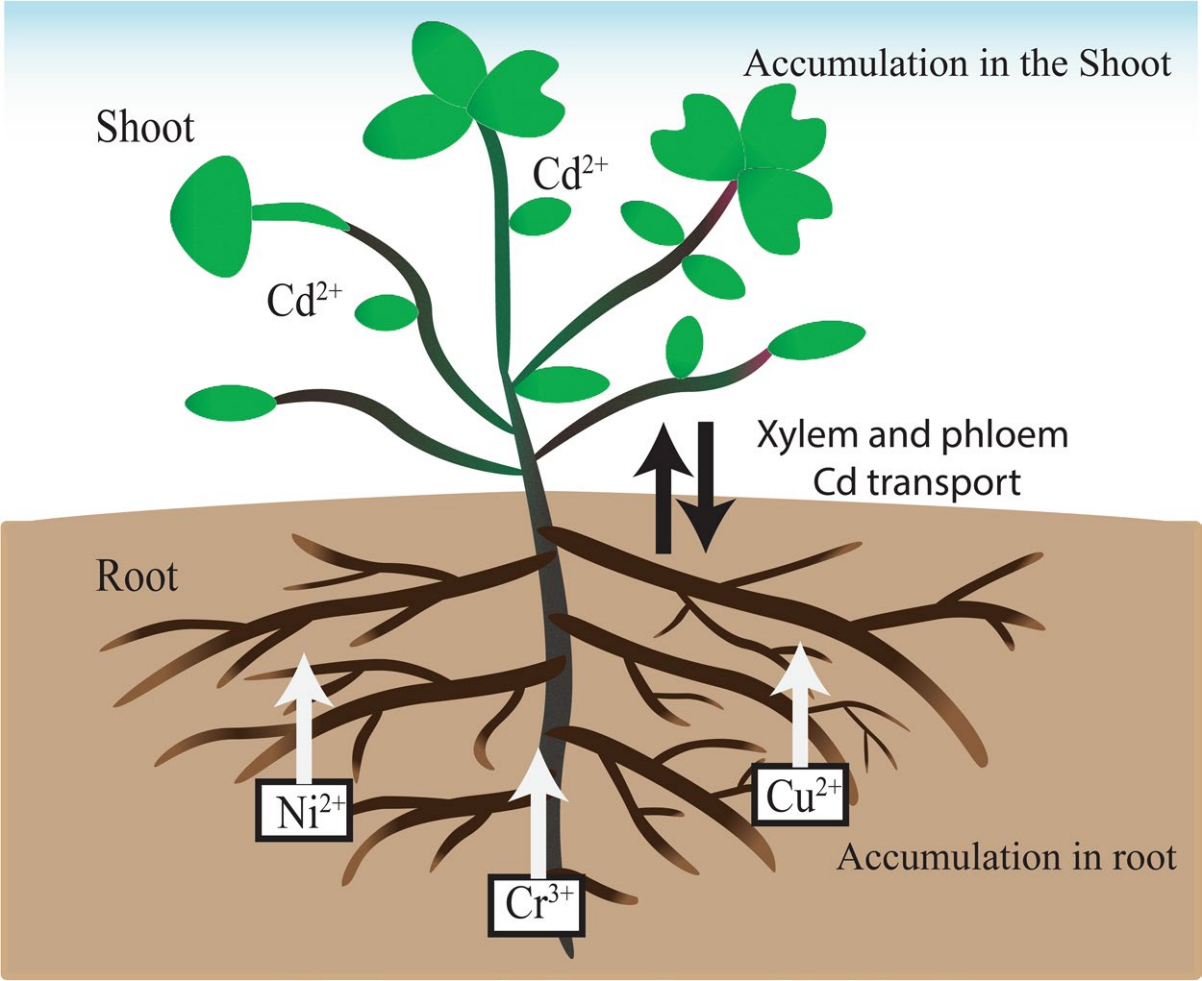
Schematic representation of Cr, Cu, Ni bioaccumulation and Cd translocation by *T. qataranse*.

FTIR results reveal that binding interaction exists with amide, hydroxyl, phosphate, and carboxyl groups. The mechanism of heavy metals removal is mostly due to ion exchange via the carboxyl groups present on the plant’s surface. For transition metals such as Cd, Cr, Cu and Ni, interaction with plant biomass largely via amino sugars^24^. Primarily, these sites are for H^+^, Na^+^, K^+^, Ca^2+^, Mg^2+^ and Fe^+^ cations. However, in the presence of metals such as Cd^2+^, Cr^3+^, Cu^2+^ and Ni^2+^ there is the tendency for substitution between metals^51^. The schematic representation of tissue-specific metal accumulation in *T. qataranse* is shown in Fig. 5. The affinity of different plant tissues towards specific metal ions depends on the available binding sites^52^. Numerous plants are known for reduced metal uptake to aerial parts^39^. The root has a vital role in plant growth and development, and therefore dictate other tissue response^53^. Under heavy metal stress in the soil, the root tissue suffers first exposure. Plant cell wall has a mechanism of exchange that fixes the heavy metal ions, thereby limiting transmission to other tissues^8^. According to the Pearson classification, Cr, Cu and Ni are on the borderline of polarizable and non-polarizable metals, whereas Cd belongs to the polarizable or soft category^54^. The translocation of Cd to the shoot may be due to its soft cationic nature. It is more likely to form stable complexes with like donors, the soft ligands, such as the amino and sulfhydryl groups. Whereas Cr, Cu and Ni affinity to root is due to their more stable complex formation with hard ligands; hydroxyl, carboxylate, carbonate, and phosphate groups. Considering Cd elemental properties and mechanism of uptake in plants, it is readily bioavailable and efficiently translocate from the roots to other parts. Substantial evidence suggests that it enters the plant via essential elements (Ca, Fe and Zn) uptake system, and that even guard cell Ca^2+^ channels are permeable to Cd^2+^ ^55^. Dalton, *et al*.^56^ refer to Cd^2+^ as “opportunistic hitchhiker.”

Therefore, metals interaction with *T. qataranse* biomass is via carboxyl and amino group. Following cationic exchange for metals via the functional groups, alteration of lignin, cellulose and protein structural moieties leads to changes in the plant’s growth pattern, photosynthetic activity and antioxidant system^24,57^. Indeed, several studies reported single and combined effects of these metals (Cd, Cr, Cu, Ni and Pb)^58–61^ on photosynthetic activity, proline content and soluble sugars. Others are increased activities of total glutathione, ascorbate, peroxidase, superoxide dismutase and catalase in plant species including *B. juncea, K. obovata, O. sativa, S. drummondii*. Therefore, *T. qataranse* response to Ba, Cd, Cr, Cu, Ni and Pb, and bioaccumulation pattern in this study is partly due to the metals antagonistic effects and antioxidant enzymatic activity.

Other metal properties presented in Table 3 contributes to their binding behaviour and strength, which in turn influence the adsorption mechanism in plant biomass. The hydrated ion radius determines hydration effects, and it is important to note that, for all metal ions, the hydrated radius is larger than the crystal radius (Table 3). Generally, an increase in hydrated radius indicates strong cationic hydration energy. Upon weak binding, hydration effect is most dominant and weakly hydrated larger ions preferentially accumulates in the interface^69^. Table 3 shows that Ni^2+^ has the lowest hydration radius, while Cd has the highest hydration radius. Although intra-particular mechanism may control adsorption, however, the structure and size of metal ions will most certainly influence ion mobility and consequently the adsorption mechanism. Hence, considering the ionic radius of Cd^2+^, Cr^3+^, Cu^2+^ and Ni^2+^ and other properties (Table 3), their adsorption means may have evolved. Indeed, a similar observation was made by Hawari *et al*.^53^ it, therefore, make sense to assume that, Ni^2+^ easily migrate and adsorb onto *T. qataranse* biomass. High electronegativity increases metal ions adsorption capacity. Cu^2+^ has a comparatively higher electronegativity (Table 3). Additionally, considering the parameter for covalent binding, which is a product of crystal radius and electronegativity Cu^2+^ has the highest value^71^. These indicate that like Ni^2+^, compared to other metals, Cu^2+^ also readily adsorb and has a strong binding strength to *T. qataranse* biomass.

**Table 3:** Characteristics of metal binding strength.

Metal	Charge, *z*^a^	Crystal radius, _crystal_^b^ (A°)	Hydrated ion radius, *r*_hyd_^c^ (A°)	Pauling electronegativity^d^	Parameter for covalent binding, x^2^ (*r*_cryst_ + 0.85)^e^(A°)
Cu	2	0.73	4.19	2	6.41
Ni	2	0.69	4.04	1.8	5.73
Cd	2	0.95	4.26	1.7	5.51
Cr	3	0.75	4.13	1.6	4.1

^a 68^, ^b 69^, ^c 70^, ^d 69^, ^e 71^.

## Conclusion

In this study, we provide evidence of some heavy metals contamination at Ras Laffan, and further show *T. qataranse*, an undershrub plant accumulates higher concentration of Cd, Cr, Cu and Ni than the soil. Results suggest that *T. qataranse* remediate Cd by phytoextraction, and Ba, Cr, Cu, Ni and Pb by phytostabilization. *T. qataranse* is edible to animals in an arid environment; its ability to stabilize toxic metals in the root and limited translocation to other plant parts restrict soil transport, prevent animal’s ingestion and consequent transmission across the food chain. Metals can accumulates in even higher concentration than shown here; hence, *T. qataranse* is a suitable candidate for toxic metals phytostabilization. However, further study on individual and combined effects of these (Cd, Cr, Cu, and Ni) will provide further insight into the plant’s phytoextraction potential, a more efficient form of phytoremediation.

## Materials and Methods

### The study site

The study site (25°50′30.43″N 51°34′34.31″E) is situated at the famous Ras Laffan Industrial area, approximately 80 km north of Doha city centre of Qatar. Characterized by low elevation and sandy soil, it is the largest site for the production of liquefied natural gas and gas to liquid. In addition to existing oil and gas refineries, Ras Laffan is home to three power generation and water desalination plants. It harbours the largest artificial port with an enclosed water area of about 4,500 hectares. Qatar University’s Department of Biological and Environmental Sciences field (25°22′21.60″N 51°29′45.28″E) is the control site for FTIR result comparison. It is a protected field, characterized by compact soil. It supports more moisture and organic matters. Vegetation type includes shrub trees, grasses and herbs, among which includes *Acacia spp*., *P. juliflora, Z. nummularia*, *S. imbricata, C. imbricata and L. shawii*. The site is used as control to compare FTIR data only. All six metals in the soil and *T. qataranse* samples from the control site were below detection limit following ICP-OES analysis.

### Soil pH, electrical conductivity (EC) and total organic carbon (TOC)

Soil physicochemical parameters were determined as follows; Soil pH using a portable digital pH meter (Mettler Toledo FE20 ATC). In order to determine the total concentration of soluble salts in the soil, electrical conductivity (EC) was also measured in dS m^−1^ using an inductive electromagnetic device (Mettler Toledo S230 SevenCompact)^62^. The estimation of total organic carbon (TOC) is by Walkley^63^. All analysis of  samples were  carried out in duplicate, and the average reported.

Total organic carbon was measured with a TOC analyser equipped with a solid sample module operated at 900 °C (Shimadzu 5050 A with SSM-5000A; Shimadzu, Kyoto, Japan). The analysis was performed according to ISO 10694 (ISO, 1995). Sample quantity was between 0.5 and 1 g and measurable range from 0.1 to 30 mg OC. Each sample was analysed in duplicate, and the average reported.

### Sample collection and laboratory processing

Sampling was performed in a grid-like pattern at every 4 meters spacing using a shovel and or auger; comprising of a whole plant and rhizosphere soil at approximately 20 centimetres deep from the surface. After that, samples were acid treated in plastic bags, transported to the laboratory for heavy metal pre-analysis treatment. Air-dried soil crushed into smaller particles, and visible plant materials, such as roots and residues removed with tweezers. Each sample was ground to a fine powder using agate mortar and pestle before digestion. Plants samples washed with tap water to remove excess soil, particularly in the root. Acid washed in 0.01% HCl and thoroughly rinsed with deionised water^64^. Following sterilisation, plant samples were separated into two parts; shoot (aboveground biomass) and root (belowground biomass). Tissue samples were air dried at room temperature for 48 hours, and later at 80 °C oven temperature for 48–72 hours. The dried root and shoot parts were then ground to a powder using a mechanical stainless steel grinder and a wooden mortar and pestle, respectively. For both soil and plant tissue samples, excellent powdered sample starting materials were obtained by sieving with a 0.25 mm diameter mesh utensil.

### Nitric acid digestion

Samples digestion were carried out using nitric acid (HNO_3_) and hydrogen peroxide (H_2_O_2_) or hydrogen fluoride (HF) for plant tissues and soil, respectively. A large capacity *Environmental Express* SC154 HotBlock ^®^, digestion system, was used as an alternating temperature following the Environmental Protection Agency (EPA) method 3050^65^. Before digestion, all vessels and glassware were acid washed and water rinsed. The analytical weighing balance was used to weigh samples to approximately 0.5 g for plant tissues (Root and shoot) and 0.25 g for soil samples, which proceeded as follows. *Soil:* About 9 mL 65% nitric acid added to each sample containing digestion vessel, gently swirled and placed onto a Hotblock system at 95 °C for 30 minutes. After 30 mins, while samples were still on the Hotblock digester, 3 mL HF added and further digest at 95 °C for another 30 minutes. Afterwards, the temperature increased to 135 °C for 1 hour and later raised to 155 °C to evaporate samples to almost dryness. Subsequently, about 3 mL HNO_3_ followed by 40 mL of de-ionised water was added and boiled until clear. Clear solutions quantitatively transferred to a 150 ml volumetric flask. After cooling, all samples made to a final volume with deionised water. *Plant tissues:* Root and shoot samples were digested as follows; 10 mL 65% HNO_3_ and 2 mL 30% H_2_O_2_ were added, gently swirled and placed onto the HotBlock digestion system. Samples heating follows at an alternating temperature of 95 °C–135 °C until clear. After cooling, clear solutions were quantitatively transferred into a 100 mL volumetric flask and made up to final volume with deionised water.

### Analysis of heavy metals

Following nitric acid digestion, samples analysed by direct injection into Inductively Coupled Plasma Optical Emission Spectrometry (ICP-OES). The concentration of six heavy metals (Ba, Pb, Ni, Cu, Cd, and Cr) quantified against National Institute of Standards and Technology (NIST) multi-element Standard Reference Materials (SRM’s); Soil 2709a and Apple leaves 1515. The choice of these metals is because of their public health risk, and evidence of occurrence in the Qatari environment documented in the literature. Metal analysis was performed on samples collected at Ras Laffan, and for FTIR analysis, soil and *T. qataranse* samples collected from Qatar University to be used as controls were also analyzed in ICP-OES.

### Fourier transformed infrared spectroscopy (FTIR)

FTIR analysis was performed according to Naumann, *et al*.^66^. Soil and *T. qataranse* tissues collected from Ras Laffan were the treatments, while soil and *T. qataranse* tissues collected from Qatar University (An area with the studied metals below detection limit) are the controls. Samples dispersed in dry KBr pellets were analyzed using FTS-135 (Bio-Rad) spectrometer; spectra data recorded within 400–4000 Cm^−1^ range. Codes were assigned to both treatments and controls as follows; Soil treatment (SoT); Root treatment (RoT); Shoot treatment (ShT); Soil control (SoC); Root control (RoC) and Shoot control (ShC).

### Statistical analysis

One-way analysis of variance (ANOVA) and least-significant-difference test were performed to evaluate statistical significance using Sigma Plot 13 software. Statistical significance was considered at *P* < 0.05. The correlation coefficients (*r*) of various metals were computed using Pearson’s regression equation at two levels (i) between metal concentration in the soil and different plant parts (root and shoot), and (ii) between metal concentration in the root and shoot.

Bioconcentration factor (BCF) computed as heavy metal accumulated in each plant tissue to that dissolved in the soil medium as shown below.

1 $${\rm{Root}}\,{\rm{bioconcentration}}\,{\rm{factor}}:\,{{\rm{BCF}}}^{{\rm{r}}}={{\rm{C}}}_{{\rm{root}}}/{{\rm{C}}}_{{\rm{soil}}}$$

2 $${\rm{Shoot}}\,{\rm{bioconcentration}}\,\mathrm{factor}:\,{{\rm{BCF}}}^{{\rm{f}}}={{\rm{C}}}_{{\rm{shoot}}}/{{\rm{C}}}_{{\rm{soil}}}$$

Translocation factor (TF) of examined heavy metals were computed using the above equations as follows^67^.

3 $${\rm{TF}}={{\rm{BCF}}}^{{\rm{shoot}}}/{{\rm{BCF}}}^{{\rm{root}}}$$

## Supplementary information

Supplemtary Figure S1
