# Supplementary material for: The assessment of cadmium, chromium, copper, and nickel tolerance and bioaccumulation by shrub plant Tetraena qataranse
Source: Sci Rep. 2019 Apr 4;9:5658. doi: 10.1038/s41598-019-42029-9 (PMC6449511; doi:10.1038/s41598-019-42029-9)
Supplement: Supplementary file 1 — Supplemtary Figure S1 [file 41598_2019_42029_MOESM1_ESM.pdf]

The assessment of cadmium, chromium, copper, and nickel tolerance and bioaccumulation by shrub plant *Tetraena qataranse*

Kamal Usman[1], Mohammad A. Al-Ghouti[1], Mohammed H. Abu-Dieyeh*[1]

[1]Department of Biological & Environmental Sciences, College of Arts & Sciences, Qatar University, Doha, Qatar.

*dandelion@qu.edu.qa

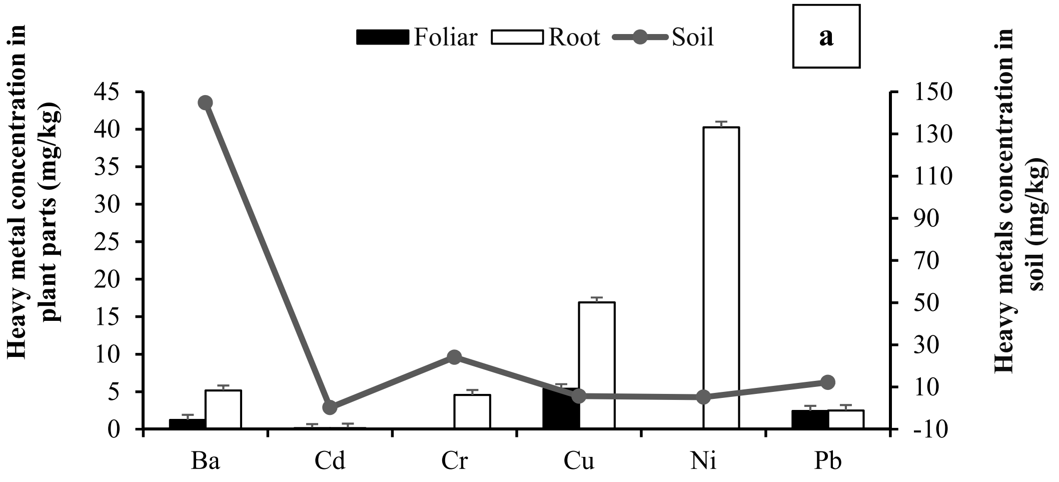

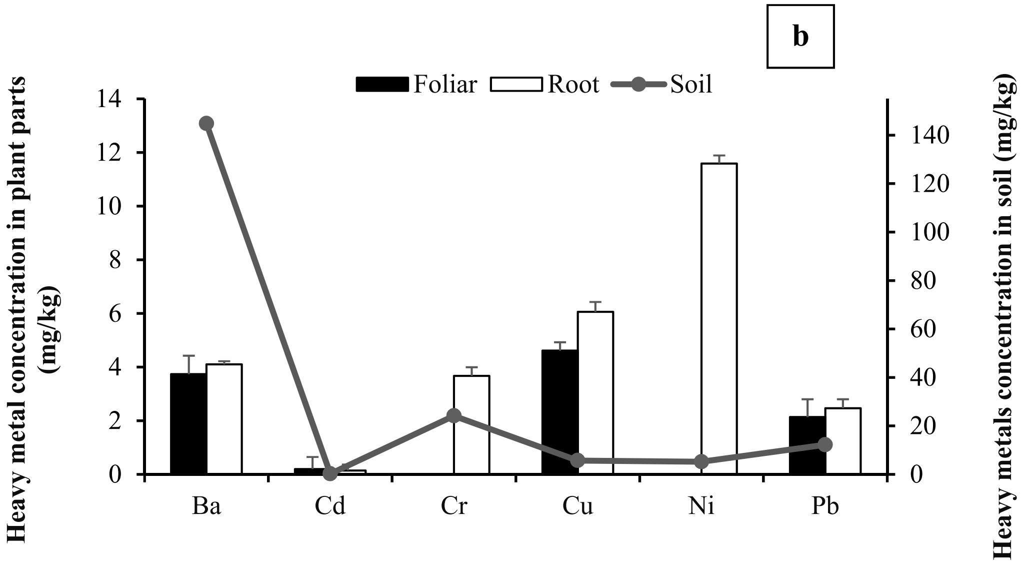

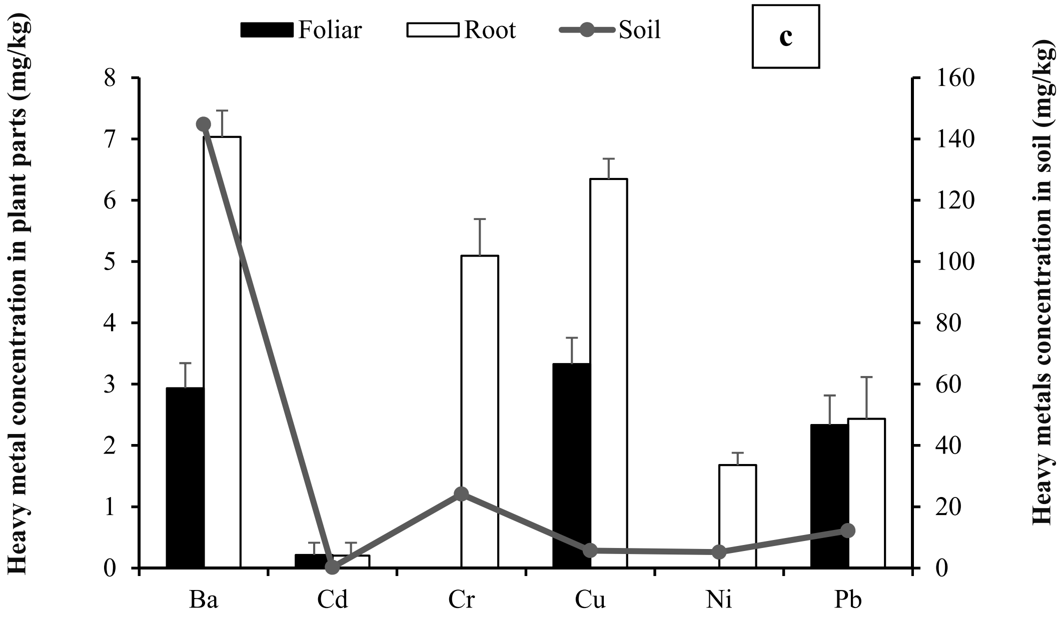

Supplemtary Figure S1. Heavy metals concentration in the soil, root and shoot of (a) *Sueada aegyptiaca (b) Salsola vermiculata* and (c) *Limonium axillare.* Mean concentration of metals are averages of five replicates (n=5) ± SEM at $P < 0.05$ level.
